# Barriers and Facilitators in the Strengthening Families Program (SFP 10–14) Implementation Process in Northeast Brazil: A Retrospective Qualitative Study

**DOI:** 10.3390/ijerph17196979

**Published:** 2020-09-24

**Authors:** Ingrid Gomes Abdala, Sheila Giardini Murta, Jordana Calil Lopes de Menezes, Larissa de Almeida Nobre-Sandoval, Maria do Socorro Mendes Gomes, Karina Damous Duailibe, Danielle Aranha Farias

**Affiliations:** 1 Institute of Psychology, University of Brasília, Brasília 70910-900, Brazil; jordanacalil@gmail.com (J.C.L.d.M.); nobre.lan@gmail.com (L.d.A.N.-S.); ninag.unb.br@gmail.com (M.d.S.M.G.); daniellearanhaf@gmail.com (D.A.F.); 2 Institute of Political Science, University of Brasília, Brasília 70910-900, Brazil; karinad210@gmail.com

**Keywords:** process evaluation, prevention, implementation, family-based intervention, program transference

## Abstract

This study analyzed contextual barriers and facilitators in the implementation of Strengthening Families Program (SFP 10–14), Brazilian version, a family-based preventive program focused on the prevention of risk behaviors for adolescent health. SFP 10–14 was implemented between 2016 and 2017 for socioeconomically vulnerable families in four Northeast Brazilian states as a tool of the National Drug Policy. A retrospective qualitative study was carried out in which 26 implementation agents participated. Data from 16 individual interviews and two group interviews were analyzed through content analysis. The most recurrent barriers were the group facilitators’ working conditions, weak municipal administration, precarious infrastructure, inadequate group facilitator training methodologies, low adherence of managers and professionals, and funding scarcity. The conditions highlighted as favorable to the implementation were proper intersectoral coordination, engagement of involved actors, awareness of public agency administrators, municipal management efficacy, and efficient family recruitment strategies. Favorable political contexts, engagement of implementation agents, and intersectoral implementation strategies were identified as central to the success of the implementation of SFP 10–14, especially in the adoption of the intervention, community mobilization, and intervention delivery stages. Further studies should combine contexts, mechanisms, and results for a broad understanding of the effectiveness of this intervention in the public sector.

## 1. Introduction

The health of Brazilian adolescents has deteriorated in recent years according to several indicators. Findings from three editions of the National School Health Survey (2009, 2012, and 2015) indicated an increase in tobacco-related product consumption (from 7.6% to 9%); fights involving a firearm (from 4% to 5.6%) or cold weapon (from 6.1% to 8.2%); unauthorized driving of motor vehicles (from 18.5% to 24.8%); and less frequent use of condoms in first intercourse (from 75.9% to 66.2%) [1]. Furthermore, the data indicated higher alcohol consumption by adolescents who reported having suffered domestic violence, skipping classes without their parents’ knowledge, parents not knowing what they did in their free time, having fewer meals with their parents, and parents not caring or caring little if they came home drunk [1]. On the other hand, living with parents, having meals together, and parental supervision were identified as protective for psychoactive substance consumption. Family supervision, as well as social determinants, such as being white, studying in a private school, and having a mother with a high school education were associated with greater use of health services. These data reveal the vulnerability to which Brazilian adolescents are exposed, especially those who are economically disadvantaged, black, less educated, and from less supportive families as well as the urgent need for intersectoral public health protection policies [1].

In 2013, Brazil’s Ministry of Health adopted internationally developed evidence-based drug abuse preventive programs focused on the school environment [2,3,4] and the family environment [5], in response to the lack of effective and scalable national preventive programs [6]. The Strengthening Families Program (SFP 10–14), or *Programa Famílias Fortes*, as it is known in Brazil, originally developed at the University of Utah in the United States [7], was one of the programs selected due to previous studies indicating its long-term effectiveness [8,9] in various countries [10,11,12,13]. The Ministry of Health decided to implement it among impoverished families with adolescents (10 through 14 years of age) who were users of “*Bolsa Família*”, a conditional cash transfer program that aimed to reduce hunger and provide access to social rights related to health and education services and to integrate adults into the job market [14]. As predicted by the resilience model and the social-ecological model, Kumpfer (2014) hypothesizes that drug abuse in adolescents from these families would be prevented by strengthening ties, cohesion, and family resilience in the family sessions; life skills in the youth sessions; and authoritative parenting style in the parent sessions. 

Between 2013 and 2017, when its implementation was driven by the Ministry of Health and the Ministry of Justice as a tool of the National Drug Policy then in force, SFP 10–14 reached 10 states in all five regions (North, Northeast, Center-west, Southeast, and South) of Brazil (Brazil, Ministry of Health, 2017). In 2015, the National Secretary of Drug Policy (Ministry of Justice) ordered a study to assess the effectiveness, social validity, and implementation quality of SFP 10–14 in Brazil. That evaluation covered the implementation in Northeast Brazil, where this study was carried out. Historically, it is the Brazilian region with the highest levels of extreme poverty (IBGE, 2017), despite efforts employed in the first decade and a half of this century to reduce social inequities [14] and broaden the coverage of basic health care services [15]. The implementation of SFP 10–14 in Northeast Brazil was shared among professionals affiliated to basic healthcare, education, and social protection services, aiming to induce intersectorality and strengthen the network of local services provided in public policies for economically disadvantaged families.

This context of vulnerability is different from the contexts of other international studies, which compare the effectiveness of local programs versus culturally adapted programs [16]. Studies clarifying how context affects the implementation process can help interpret the findings of effectiveness studies [17] as well as supply inputs for large-scale implementation, a crucial item in public policies, aiming for the ethical and efficient use of public resources. The context, far from being merely background, refers to a set of characteristics and circumstances of dynamic and unique factors into which the implementation is inserted, and which interacts, influences, modifies, facilitates, or impedes the intervention and its implementation [18]. According to the Context and Implementation of Complex Interventions (CICI) model proposed by Pfadenhauer et al. (2017), the context includes not only aspects of the physical space, but roles, interactions, and relationships at several levels: geographical, epidemiological, sociocultural, socioeconomic, ethical, legal, and political. These, in their turn, interact with the intervention’s implementation, e.g., the program’s action theory, implementation strategies, implementation agents, and the intervention itself. 

Even though SFP 10–14 has been culturally adapted to different contexts, including European [19], Latin [20] and Asian [21] countries, paradoxically, previous studies of SFP 10–14 have omitted analyses of how context (broadly defined) affects implementation, as proposed by Pfadenhauer et al. (2017). An integrative literature review focused on SFP 10–14 evaluation studies identified that, out of 61 articles, only 18% assessed SFP 10–14’s implementation dimensions [22]. The studies primarily covered fidelity analysis, and less frequently, adaptations, economic cost, dose received, engagement, retention and barriers, and implementation facilitators. The findings indicated that SFP 10–14 had its implementation facilitated as much by offering childcare for children under 10 years as when it had experienced group leaders who were bilingual and culturally competent [23]. On the other hand, implementation impairment occurred due to the leaders’ difficulty in planning the sessions beforehand [24], low adherence from the families [25], and insufficient engagement from the school [23]. Thus, there are still unknown contextual barriers and facilitators for the implementation of SFP 10–14.

This study was guided by the following research question: How did context influence the SFP 10–14 preimplementation and implementation process in Northeast Brazil? The aim of this retrospective qualitative study was to examine contextual factors that acted as barriers or facilitators throughout the preimplementation and implementation stages of SFP 10–14 in Northeastern Brazil. Implementation is understood as the delivery of the intervention, while preimplementation refers to the previous exploratory and preparatory stages of the intervention’s implementation itself, both of which are affected by multiple contextual factors [26,27]. While the intervention delivery (implementation) refers to offering seven regular meetings for families and adolescents, the exploratory and preparatory stages (preimplementation) include tasks such as the adoption of the intervention in the local context, the building of community coalitions to offer the intervention, training the implementation team, family recruitment, and institutional arrangements for delivery of the intervention.

It is expected that this study, through analyzing the implementation context of a culturally adapted program for a Latin American country, can expand existing evidence regarding contextual barriers and facilitators when implementing family preventive programs in resource-poor settings. Furthermore, the findings of this study may enhance intervention scalability planning, indicating potentially successful or risky implementation routes as part of a public policy system. Finally, it is expected that the results will contribute to some extent to the generation of hypotheses that facilitate understanding the effectiveness findings of parental preventive programs transplanted to regions other than those in which they were created [16].

## 2. Materials and Methods 

### 2.1. Brazilian SFP 10–14 Implementation Context

**Figure 1:**
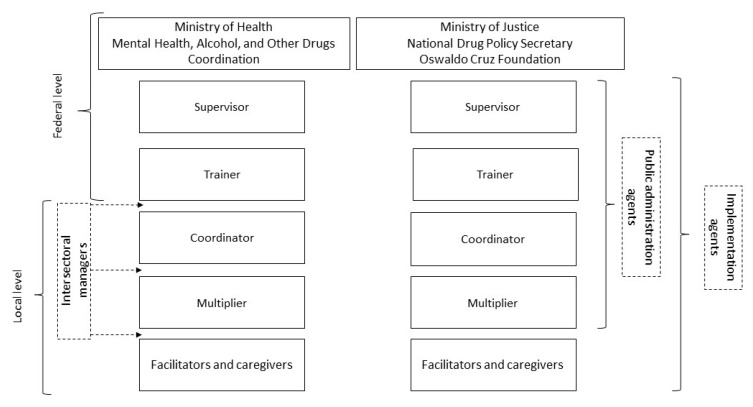
The organizational flow of the implementation of the Strengthening Families Program (SFP) 10–14 in Brazil.

The implementation of SFP 10–14 in Brazil was structured on the following organizational flow (Figure 1): (i) Ministry of Justice and Ministry of Health: federal entities responsible for nationwide implementation management, with the Ministry of Health having been responsible for SPF 10–14’s adoption and cultural adaptation to Brazil; (ii) National Drug Policy Secretary, Coordination of Mental Health, Alcohol and Other Drugs, and the Oswaldo Cruz Foundation: entities responsible for program coordination and implementation in the states; (iii) federal supervisors: workers who played a fundamental role in prevention policy promotion and local manager awareness-raising for the adoption of SFP 10–14 and were also responsible for supervising the work of the federal trainer; (iv) federal educators: workers who performed the study and selection of public social assistance, health care, education, and similar services in the municipalities, jointly with local managers, to implement the program and who were also responsible for the training of the program multipliers and group facilitators, supervising them throughout the implementation; (v) local coordinators: professionals’ knowledge regarding the realities of municipalities and who coordinated intersectoral services and built organizational and community support for the execution of SFP 10–14; (vi) multipliers: in charge of mediating the relationship between the municipality’s local coordinator, federal management (via the federal trainer), and the SFP 10–14 group facilitators and monitoring, supervising, and orienting the local group facilitators and, often in addition to their assigned function, exercising the function of group facilitator; (vii) group facilitators: these workers facilitated the intervention sessions, with at least one group facilitator for the parent groups and two for the adolescent ones; and (viii) childcare providers: workers who cared for and played with children under 10 years of age not attending the intervention.

### 2.2. Design and Participants

This is a retrospective qualitative study. An intentional sample was used, comprising 26 implementation agents who participated in individual interviews (16 participants) and two group interviews (10 participants). The participants acted directly in the program implementation in four states: Ceará, Pernambuco, Rio Grande do Norte and Sergipe. The inclusion criteria was participation in at least one implementation cycle of SFP 10–14 as a program implementation manager. The number of implementation cycles varied between one and two cycles when collecting data in different states. Six participants were reached at the end of the first implementation cycle, while twenty were reached at the end of the second implementation cycle. 

The sample comprised two federal supervisors, four federal educators, seven articulators, and thirteen program multipliers, distributed evenly throughout the four states. The participating professionals come from the fields: psychology (12), social assistance (5), nursing (2), pedagogy (2), social science (1), physical education (1), history (1), occupational therapy (1), and biological sciences (1) and had an average age of 36 years. All the coordinators and multipliers worked in activities beyond the implementation of the program, for instance: as a psychologist for the Social Assistance Reference Centers (SARC), as a nurse for Basic Healthcare Units, or as a teacher in a community school.

### 2.3. Instrument

An interview guide based on SFP 10–14 action theory in Brazil was used to conduct the interviews and focus groups. The action theory is conceptually defined as the program’s theory of change and seeks to explain the causal implementation mechanisms [28]. Thus, a theory of action explains how an intervention works and how it contributes to a chain of results that produce the intended or actual impacts. In this study, the action theory was defined operationally as the preimplementation and implementation stages of SFP 10–14, their interconnection over time, and the expected outcomes for the success of the implementation process’s next steps, ultimately impacting its effectiveness. Table 1 presents the SFP 10–14 action theory stages and their expected results, defined exclusively by the authors for Brazilian context, previously identified in three focus group sessions with the federal coordination and supervisors at the beginning of this study. The interview guide investigates contextual aspects that participants considered either barriers or facilitators in the program implementation process in each action theory step via a total of seven questions (Table 1). For example, the first question was: “*Regarding the negotiation between local and federal government to bring the SFP 10–14 to [name of the city where the participant worked], what facilitated the process and what were the barriers?”* The second question was: “*Regarding the local coordination of the SFP 10–14 in [name of the city where the participant worked], what facilitated the process and what were the barriers?*” and so forth. 

### 2.4. Strengthening Families Program (10–14)

SFP 10–14 comprises seven two-hour long weekly sessions and four booster sessions, with the participation of children and adolescents 10 to 14 years old and their parents and/or legal guardians. In the first hour, children/adolescents and parents attend separate meetings and participate in age-appropriate activities. The modules for children/adolescents aim to improve self-efficacy in dealing with stress and peer pressure. The modules for parents stimulate the development of an authoritative parenting style, matching high levels of demandingness (control and supervision) and positive affect (involvement and warmth). In the second hour, the families reunite, and the activities address communication patterns and strengthening of family cohesion [29].

SPF 10–14 has an implementation manual, translated and adapted for Brazil [30]. The procedures and materials were analyzed in a pilot study in 2013 in the Federal District to assess needs for cultural adaptation [5]. This was followed by cultural adaptation in its superficial structure before its dissemination to other Brazilian regions [31]. The adaptations were intended to provide greater customization to the local culture and included changes in the names of activities, welcoming procedures, dubbing the videos in Portuguese, new illustrations, adding a facilitator to the youth session in order to facilitate the management of the group, reduce the duration of facilitators’ training from four to three days and addition of a guide for facilitators with tips to facilitate the execution of activities. Changes in the deep structure of the program, such as changes in objectives, content, sequence and format of the meetings, were not carried out, and the core components of the intervention were preserved. The objectives, activities, and materials are described in detail and the activities are timed. Audio and visual materials are used, displaying scenes of family interaction, to offer a reference for competence in communication, emotional regulation, and collaborative resolution of family problems. The intervention prescribes offering meals to participants, based on the evidence that family meals strengthen family cohesion [32]. Furthermore, it prescribes childcare for children under 10 to boost participant retention.

### 2.5. Data Collection Procedures

Both individual and group interviews were carried out by trained researchers. The researchers contacted all participants by telephone, between one and three months after the implementation of SFP 10–14, and presented the study objective, the expected length of the activity, the confidentiality of information, and the voluntary nature of participating in the study. At the convenience of the participants, it was agreed to conduct the interview individually or in groups. The individual interviews were conducted by telephone. An image in PDF was sent via email with the seven stages of the SFP 10–14 action theory, described in Table 1, to guide the interview. The individual interviews lasted 40 min on average. The group interviews took place in face-to-face meetings, at a university and at a SARC, and lasted one and a half hours on average. All interviews were carried out between June and August 2017. To facilitate the discussion, the same image of the action theory stages from the telephone interviews was given out in the group interviews. Both individual and group interviews were audio recorded and then transcribed by the research team. Interviews were discontinued when data saturation was reached. 

### 2.6. Data Analysis Procedures

**Figure 2:**
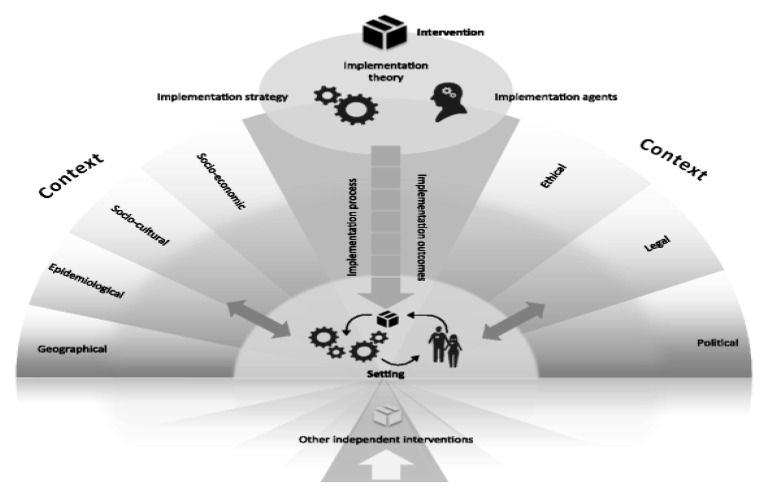
The Context and Implementation of Complex Interventions (CICI) framework. Reproduced with permission from [18], [Implementation Science]; published by [Springer Nature], [2017].

Content analysis combining inductive and deductive categorization with frequency counting of categories was performed. This content analysis modality was chosen because it allows organizing the data with and without previous categories, in order to accommodate the richness of the data and, also, for allowing to compare the magnitude or relevance of the categories, based on their numerical expression. Firstly, preexploration of the transcribed material was carried out through floating readings of the interviews corpus. The objective of this stage was to select units of analysis that answered the research question. After analyzing 18 transcriptions (16 interviews and two group interviews), a total of 448 analysis units were obtained. Secondly, themes were identified and defined inductively, according to the categories developed for this study (Table 2). These categories emerged after successive readings of the data, in light of the research question. Thirdly, data were categorized deductively accordingly to the CICI framework proposed by Pfadenhauer et al. [18] (Figure 2). Context domains referenced by interviewees were geographic, sociocultural, socioeconomic and political; implementation domains cited were implementation process, strategies, and agents, as defined in Table 3. Fourthly, the frequency of each theme was counted. Frequency counting refers to the number of times that different negative (barriers) or positive (facilitators) themes were reported, rather than the number of people who reported them.

Categorization of the data was performed by independent coders, who had had expertise in process evaluation methods of health interventions. All the categories found were revised twice. Initially, revision was done by the first author who categorized it, correcting any mistakes of synthesis and categorization; and then by the third author, for a second verification and correction of mistakes and biases in data interpretation. Disagreements among the coders were solved by the second author, who acted as a judge. Member checking was used to assess the data reliability. The category system was discussed with the group facilitators from the four states, who assessed the credibility and accuracy of the categories and subcategories. No divergence was detected. All data was imported into NVivo11.0. The categories with frequency lower than three reports were suppressed.

## 3. Results

The results indicate a slightly higher number of barriers than facilitators. Out of the 448 units of analysis identified in the implementation process of SFP 10–14, 234 were barriers (52%) and 214 facilitators (48%). Table 4 and Table 5 describe the distribution of facilitators and barriers and their frequencies in each action theory stage. Reports were most concentrated in the delivery stage (26%), followed by mobilization (21%) and negotiation (14%), while the fewest number of analyzed reports were classified as coordination (5%), training (10%), recruitment (11%), and organization (13%).

### 3.1. Negotiation for Local Adoption of SFP 10–14

#### 3.1.1. Barriers in Negotiation

The greatest obstacles described regarded municipality management, public agency administrators’ awareness and intersectorality. Reports noted that the administration was not proactive in facilitating the program adoption and delivery process, for example: (i) bureaucracies for releasing professionals (to work in the implementation), for public service exam authorization, and for supplying transportation to operationalize the distribution of snacks; (ii) lack of knowledge about local context on the part of managers; (iii) difficulties in managing intersectoral policies before the adoption of SFP 10–14; (iv) delays in the payment of professionals; and (v) difficulties integrating SFP 10–14 actions into the SARC routine.

Public agency administrators with low awareness acted as barriers because they did not promote the adoption of the prevention policy associated with SFP 10–14. Intersectorality was noticed as a barrier in two aspects: (i) when it did not exist, the lack hindered the implementation; and (ii) when it existed, however, the professionals listed difficulties in finding compatible schedules between the group facilitators from different services. SFP 10–14 implementation, which was intersectoral, demanded the existence of good coalitions between intersectoral networks. A report that exemplified the lack of awareness of public administration agents acting as a barrier was:

“(…) there were other managers who had never participated in the process. “Oh, I agree”, but you agree and that is not the point. Perhaps some managers lacked commitment. As if it were “let the technicians take care of that”. And that doesn’t fulfill our needs, like a providing snacks, which depends on authorization from the top manager, that is hard to get. Since, we don’t often get it, everyone just do what they can.”

#### 3.1.2. Facilitators in Negotiation

A culture of prevention, awareness of public agency administrators, intersectorality, and professionals’ adherence were highlighted as facilitators of the negotiation between federal and municipal levels for the adoption of SFP 10–14 by municipalities. On a local level, prior existence of high-level managers and governmental institutions that understood prevention policies as a priority, facilitated the implementation of SFP 10–14, this being more often the case in the initial stage of its adoption. When the program was well-received by the public agency administrators starting with the negotiation stage, the subsequent stages were facilitated, leading to improved communication between these administrators and other professionals and simplifying group facilitators’ release for participating in the delivery of SFP 10–14. Previous experience with SFP 10–14 in other implementation cycles, or with other evidence-based programs, facilitated the professionals’ adherence to the program as well. 

Public agency administrators’ awareness was highlighted as a facilitating element. Its major recurrences were in the negotiation and mobilization stages. However, according to reports, the impact of public agency administrators’ awareness reverberated across all other stages. In addition, the existence of well-structured intersectoral coalitions was stressed as the most facilitative element of all. Intersectorality was highlighted in the phases of negotiation for local adoption of SFP 10–14 and session organization. Network coalitions allowed the exchange of materials and tools necessary for the program’s delivery, as did the offer of high-quality snacks. The sensitization of public administration agents and the intersectorality as facilitating elements can be seen in:

“Receive support from top managements helps a lot. From the secretary, from the mayor, (…) if they are not at the forefront, they should endorse and designate people to take care of it. If technicians have this endorsement from higher managers, the program happens, if it doesn’t, it doesn’t happen. So, in departments where the manager was more committed, we managed to do everything more easily. In our case, it was easy, because the staff was really involved, both the manager and the local coordinator.”

### 3.2. Local Coordination

#### 3.2.1. Barriers in Local Coordination

In the local coordination stage, municipal administration is again seen as the primary barrier. In addition to this, a lack of prevention culture and minimal assistance from the federal supervisors were likewise recurrent. The absence of a prevention culture had repercussions on the initial stages, from the decision not to adopt the program by public agencies administrators, through departments such as health and education declining the program, to the family recruitment stage. Furthermore, some professionals reported difficulties in spreading prevention ideals, because the daily routine was focused on urgent demands. The following report exemplifies the difficulty of professionals in understanding the objectives of the program, which theoretically are aligned with the prevention work carried out at SARC.

“For the SFP 10–14 to enter the services, there was a great resistance from the equipment professionals themselves, because it was something new and people thought they were getting more work, a demand for more work. Even the staff from the Ministry (of Health) and National Drug Policy Secretary came to provide explanations about the program. We were called for meetings to understand a little more about this program and how it would be executed. So, it would have been nice if there were always conversations and dialogues between us and the secretariat regarding how the program would be executed.”

#### 3.2.2. Facilitators in Local Coordination

Regarding facilitators, the proper choice of public services to offer the implementation deserves attention. Proper choices were a service in which all group facilitators are employees of that service and there is no need for displacement (which could make intersectoral coordination and logistics more difficult); taking in account the available professionals’ abilities to deliver the program, such as group management experience; accessibility for families to the service where the program was delivered; and knowledge about community profile prior to site selection. The following report demonstrates how these agents’ awareness facilitated the planning at this stage.

“In the municipalities that ran (the program) (...) we managed to set up good relationships with the secretaries that endorsed the local coordinator to execute and deliberate on the issue. So, it was not necessary to have to take all the information through the municipal secretary to do something, (...) received carte blanche to deliberate. (...) We believe that this was remarkably good.”

### 3.3. Service Mobilization

#### 3.3.1. Barriers in Service Mobilization

Professionals’ poor working conditions were repeatedly cited as a barrier to service mobilization and echoed down to team training and SFP 10–14 delivery stages. Most cited were the professionals’ workload, due to participating in both the team training and program implementation; the low number of employees working in the services; and the need for many professionals for the implementation and a lack of time within the work day. Difficulties imposed by municipal management in service mobilization were also reported, such as not allowing the group facilitators to perform SFP 10–14 activities as a SARC routine and late payment.

Failures were observed as well regarding the professionals’ awareness of the time requirements for participating in the training and implementing the program. Reports identified a lack of information about the true complexity of implementing SFP 10–14 before the professionals decided to participate in the program implementation team training. Managers confirmed that quitting was common during or shortly after completing it. This occurred because only during training did the professionals realize the incompatibility between the work necessary for program implementation and the work they already had. The following report points out reasons for the lack of adherence by professionals.

“(…) because of the simultaneous activities we did not always have physical space available for the activities. We had to disturb the service dynamics to carry out the program, which we did, but we knew we were holding up routine activities. That is the reason why service providers are resistant to the program, and we understand it.”

#### 3.3.2. Facilitators in Service Mobilization

Public agencies administrators who had their awareness raised by the program’s arrival acted significantly by doing the same for the implementation professionals. The reduction of bureaucratic hurdles, a consequence of having aware managers, facilitated the engagement and adherence of professionals in the team training stage. The occurrence of this gradual awareness-raising, following the hierarchical flow of the service management was reported as a facilitator. This is not the only or the best way to communicate, it just portrays an effective communication channel in program implementation. Additionally, manager awareness eased the release of professionals to work on the program.

Even though municipal management and intersectorality played mainly as barriers, they were also cast as facilitators. The existence of organized municipal management, prior to the program’s arrival, acted positively in its implementation. Such management included managing people in their activities, decentralizing power, and redirecting financial resources. The engagement of professionals acting as facilitators in this stage can be identified in:

“In fact, the secretariat of health could not provide enough material necessary for executing the SFP 10–14, such as cardboards, to everyone. There is a list, from the three departments (Health, Education, Social Assistance), that was shared to check what each department could provide. When they contributed, we put everything together and distributed it to the regions.”

### 3.4. Team Training

#### 3.4.1. Barriers in Team Training

The selection of training methodology for the group facilitators, multipliers, and coordinators was a strategy that simultaneously helped and hindered program implementation. There were conflicting opinions among these agents regarding (i) the shortening of the training course from three to two days; and (ii) the inclusion of broader themes that discussed principles of prevention policies and evidence-based programs but would reduce the time for practicing the program activities to be offered. 

While for some interviewees the reduction in time made it difficult to understand the activities—as it was not possible to practice them—for others it made the training less tiresome and more productive. For some agents, the introduction of broader themes reduced the training time for the activities of the SFP 10–14, which hampered transporting knowledge acquired in training to the local reality and caused the perception that they were less prepared for adaptations at execution time. However, for others, the insertion and discussion of these themes were fruitful for understanding the program’s essence, which facilitated the necessary “in-flight” adaptations. The following report demonstrates the interviewees’ point of view, who would prefer a longer training:

“It could be expanded (the training hours). It is because there are many activities to be developed and we do not see all the activities during training”.

As previously discussed, the professionals’ working conditions also had a negative impact on team training, especially when it impeded participation in the training due to workload. The precariousness of the working conditions and low adherence of professionals and managers involved in the implementation were also listed as barriers in this stage.

#### 3.4.2. Facilitators in Team Training

The methodologies used by federal educators in the group facilitator, multiplier, and coordinator training also deserve to be highlighted as facilitative. As an example, clear and attractive information transfer, which allowed understanding the program’s essence, even if the training did not go through all the activities proposed in the program guidebook, was cited. The following report illustrates how appropriate the methodology used was.

“There was no way for her (federal trainer) to apply all the activities in the training. What was applied served us as starting point. I think that what was explained in the training was extremely important to guide us in adapting it in different scenarios.”

### 3.5. Family Recruitment

#### 3.5.1. Barriers in Family Recruitment

Implementation strategies continue to play a fundamental role in the selection of families. Barriers in family selection and invitation have two primary sources: (i) families not meeting the program’s prerequisites, such as the ability to read and write and (ii) lack of clarity on the part of the federal supervisors regarding the target audience, consequently creating recruitment difficulties for the multipliers and group facilitators. Discrepancies were reported between the proposed target audience profile and that of the community, for example, being able to read and write or not already being a drug user. In addition, it was reported that group facilitators had difficulty denying participation to families already being assisted by a local service who did not meet all of the program’s prerequisites. 

The difficulty of recruiting the proper profile caused, according to the managers, disengagement by the professionals. This, along with the families’ incompatible schedule with the program’s, fed the barriers to family selection. The managers report that matching the community’s family profile to the choice of service to execute the program is necessary. If the community receiving SFP 10–14 is too precarious, or if the users of the services are already abusing alcohol and other drugs, then the professionals have a hard time selecting families. The following report describes the professionals’ difficulties in selecting families to participate in the program.

“(…) we knew teenagers and children that we believed should be part of the SFP 10–14, but the program was really strict with the age limit. Leaving out a nine-year-old child or a fifteen-year-old teenager who wanted to participate was a problem, however they were technically ineligible and we couldn’t take them in.”

#### 3.5.2. Facilitators in Family Recruitment

Several strategies were used in the advertising to and selection of families. The most common were actively seeking families within the community; verifying that the families had the proper profile; advertising SFP 10–14 in a youth-attractive way; mentioning the snacks and freebies in the advertisement; word-of-mouth advertising by families who had participated in prior cycles; and inviting families individually rather than in groups. Using two or more of the cited strategies was common. Although the advertisement of SFP 10–14 by former participant families was not an approach foreseen by the group facilitators, it was considered a positive one.

The strategies for retaining families, which as a consequence increased the families’ adherence to the program, also facilitated this stage. Examples of such strategies were: identifying what the families consider attractive in the program and highlighting these aspects to them in the family invitation and awareness meeting; weekly contact with the family, repeating the invitation before each meeting; in the invitation and awareness meeting, employing a methodology similar to that used in meetings and follow-ups for people with difficulty writing. Furthermore, the existence of a prior relationship between the service and the community facilitated family outreach and indicated greater engagement on their part. One of the family maintenance strategies used by professionals as a facilitating element can be seen in:

“(...) in our group there were several mothers who couldn’t read or write. We were afraid that when we handed up the brochures, they would feel embarrassed. So, since the first meeting, we welcomed them warmly so that they would not feel that way for not being able to read.”

### 3.6. Planning of Implementation Logistics 

#### 3.6.1. Barriers in Planning of Implementation Logistics 

Insufficient financial resources and infrastructure problems were the main obstacles in the stage of organizing the venues, group schedules, and materials. Common difficulties faced were the location’s room infrastructure and material resources being incompatible with SFP 10–14’s requests; unavailability of audio and video (TV, DVD, telephone); lack of a space for children under 10 years of age; rooms unavailable for the implementation, causing interruption of other activities of the service during program delivery and funding cuts for snacks. 

Another item that hampered implementation was the (in)compatibility of families’ schedules with the meeting schedule. Families preferred late afternoon meetings, which is the usual service closing time. The difficulty in finding schedule compatibility is evident in this comment:

“(...) we initially thought and still think that there is a big issue with making workers’ schedules compatible with the families’ schedules for the program’s activities. We do not want to overwork our professionals. This remains a considerable barrier.”

#### 3.6.2. Facilitators in Planning of Implementation Logistics

Planning prior to the program’s start was the most facilitating strategy in the organization stage. When well-executed, planning improved program delivery; the professionals reported feeling more prepared to handle the activities and more relaxed about the availability of the implementation material needed. Intersectorality was considered a facilitator in this stage as well. It is strongly related to the already-existing good coordination between the intersectoral networks, the quality of communication between the professionals, and their adherence to the program’s implementation. The municipalities where the networks coordinated to implement the program were able to overcome barriers due to infrastructure shortfalls, facilitating program organization. As an example, cars were used to transport audio and video equipment between services. The following report demonstrates how positive the engagement of professionals was in solving problems during the planning of the program.

“(…) the team’s engagement was the main facilitator. No worker has ever been absent nor delayed. Besides, we talked about everything, if we identified a problem, we talked about it and got it solved.”

### 3.7. SFP 10–14 Delivery

#### 3.7.1. Barriers in the Delivery of SFP 10–14

The difficulties encountered in the initial stages had an impact throughout the program, up to and including the final stage of program delivery. Lack of engagement by managers, precarious working conditions of professionals, and methodological mistakes in training were the main barriers in SFP 10–14’s delivery.

Precarious local infrastructure, low budget, and incompatibility between SFP 10–14 and the population attended by the service, with lower education levels, also had a negative impact on this stage. Such incompatibilities made it difficult to choose the participants and to engage them. The managers reported it was hard to carry out the weekly session planning since this activity requires the availability of transportation for the group facilitators (infrastructure), the release of professionals for this activity (adherence and awareness of management agents), and existence in the municipality of prior good communication between networks and professionals (relationship between professionals and intersectorality). Finally, they list, with less frequency, barriers resulting from the absence of a prevention culture and local strikes. The following report summarizes how the professionals’ precarious working conditions made this stage difficult.

“It is too much (...) from a single sector (not intersectoral). The program is too heavy for the service. This year we had a lot of work on trying to make the health department and the social assistance department work together. We hoped joining forces would make the implementation not so burdening. And it was not easy task, but we got more material and more human resources by bringing all departments together.”

#### 3.7.2. Facilitators in the Delivery of SFP 10–14

The perception of the effects on the families was an aspect understood only as a facilitator in the engagement of professionals. This means that the more families demonstrated the positive effects of the program or reported it, the more the professionals got involved in the program’s delivery, playing an ever more committed role. The following report exemplifies how the perception of the effects of SFP 10–14 on families was positive to engage professionals in its execution.

“(...) speaking of my team, what convinced us during the meetings was really what the families were bringing to us as a result, strengthening their bonds, and we clearly saw that little by little it was happening. We saw that it was really working for the group. That is what convinced us. I speak for myself that I was very resistant after the first meeting, I was not very fond of the idea. I didn’t like being pushed to do this, but after the second and third meetings when we heard feedback from the families, and we started to get excited.”

Despite reports of weak support from federal trainers, there was a greater frequency of reports affirming their presence and the benefits that came with it. This shows the importance of the weekly clarification of doubts that arose during the program delivery stage. In addition, the prior presence of high-level local managers and of governmental institutions that understood prevention policies as a priority facilitated the program implementation, which was more frequent in the initial stage of adoption and during the program’s execution. When receptiveness was present in the negotiation stage, the following stages were facilitated because of greater manager awareness, improving communication between them and the professionals and reducing hurdles to releasing the group facilitators. Furthermore, experience with SFP 10–14 in previous implementation cycles facilitated the professionals’ adherence to the program. The professionals’ joint operation was indicated as essential after having acquired experience with the program in previous years.

### 3.8. Barriers and Facilitators in the Implementation of SFP 10–14 in Brazil: A Synthesis

**Figure 3:**
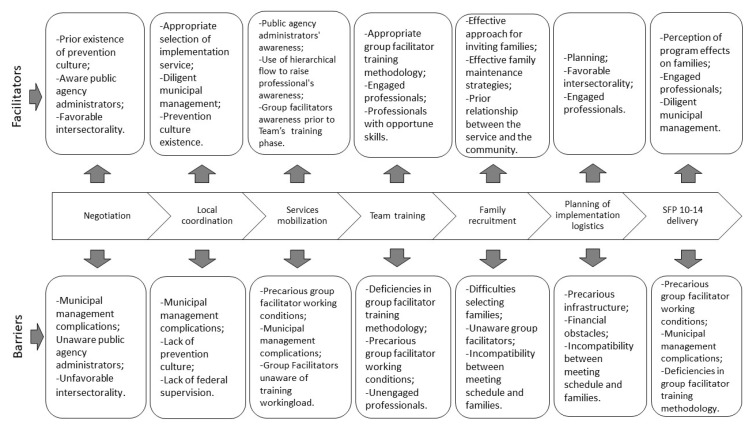
The most recurrent barriers and facilitators over the course of the preimplementation and implementation process of SFP 10–14 in Brazil.

Figure 3 summarizes the most recurrent barriers and facilitators throughout the preimplementation and implementation stages of SFP 10–14 in the analyzed Brazilian states. Note the transversality of management conditions, awareness of key actors, and quality of the implementation strategies.

The inductive and deductive systems of analysis were combined, and this process resulted in the categorization proposed in Table 6.

**Figure 4:**
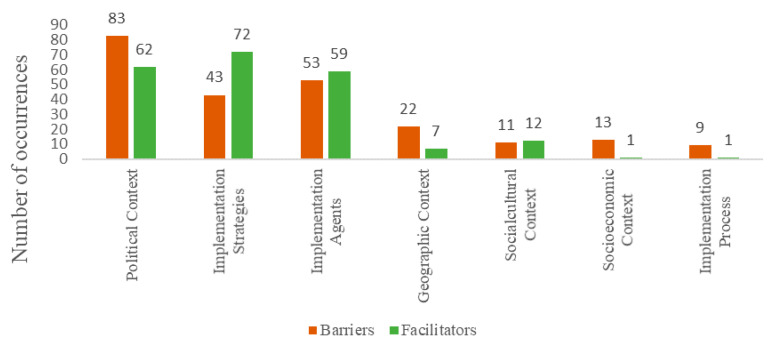
Total of barriers and facilitators in the SFP 10–14 implementation process in Brazil according to the Context and Implementation of Complex Interventions model.

Data analysis according to the categories of the Context and Implementation of Complex Interventions model [18] is described in Figure 4. The political context, implementation agents, and implementation strategies were most influential in the implementation of SFP 10–14, while the geographical, social-cultural, social-economic contexts and implementation process were mentioned less frequently.

## 4. Discussion

The present study was guided by the following research question: how did the context influence the preimplementation and implementation of SFP 10–14 in Northeast Brazil? The analysis of interviews revealed a broad range of contextual factors that impeded and facilitated the implementation concentrated in the delivery, negotiation, and mobilization phases, with barriers having been reported slightly more than facilitators. The reports indicated that the SFP 10–14 implementation process was facilitated, primarily, when the implementation agents were engaged from the program’s adoption until its delivery and, secondarily, when the municipality, even a highly economically vulnerable one, was organized ahead of time to host the program, with appropriate infrastructure allocated, elevated planning capacity, and effective intersectoral coordination. This organization was enhanced in locations that had previously implemented policies of a preventive nature, such as the SFP 10–14 itself, or when the professionals and managers had chosen to incorporate prevention science and evidence-based science goals into practices.

The findings point to the political context, the implementation strategies, and the implementation agents as having substantially impacted the implementation. As such, it should be noted that the majority of incentives considered helpful for the implementation involved better communication between the intersectoral networks, greater involvement of implementation agents in prevention policy and the program, and better understanding of the primary prevention objective and the intervention itself. These last aspects are consistent with findings of an evaluation of the SFP 10–14 implementation process in Wales, United Kingdom, which also identified greater commitment to the intervention by professionals who understood its mechanisms and content as facilitating the implementation [33]. Additionally, positive strategies were developed from earlier experience implementing SFP 10–14 or from prior knowledge of prevention. Altogether, these initiatives seem to favor the construction of a culture of prevention, which encompasses gradual and procedural learning practices and results in important contextual innovation.

Exercising preventive practices in the daily routine of a service and promoting a prevention culture go hand in hand [34]. The more the services engage in preventive activities or programs that reduce health risks, the more prevention is spread. In this sense, it can be presumed that the simple existence of a prevention program like SFP 10–14 in services plays a primary role in stimulating a prevention culture in the public service [35]. In Brazil, this would imply structural paradigm shifts in which urgency would no longer be the only item in the policy agenda but would share space with prevention, particularly by public sector decision-makers. These actors play a central role in maximizing engagement of implementation teams and in the feasibility of necessary implementation processes for a preventive delivery system. Added to this is the importance of continuous education to strengthen the prevention culture [36].

While, on the one hand, the political context turned out to be key to the success of the preimplementation and implementation, on the other, the geographic, sociocultural, socioeconomic, and aspects of the implementation process were shown to have little influence on the SFP 10–14 implementation path. A precarious infrastructure and limited financial resources were relatively low-impact challenges for the implementation, even though program delivery occurred in one of the poorest regions of Brazil. Similar findings were uncovered in a systematic review that synthesized recommendations derived from 31 evidence-based program evaluations for solving problems related to public health policies [37]. Ubbink et al. (2013) identified (i) the predominance of the impact of the political context, which involves coordinating intersectoral networks, managing people, distributing power, and administering financial resources and (ii) centrality of communication, engagement, and raising the awareness of institutions and of management and implementation agents in the program’s execution. These results do not indicate that poor financing and infrastructure deficiencies should be ignored, but permit the conclusion that many problems could be solved from other directions, such as improving management, implementation agent engagement, and promoting integrated and intersectoral policies that enable favorable political contexts.

The political context brought the greatest number of implementation barriers, the most cited being the management decision-making concerning the administration of people and financial resources and the existence of coalitions between networks, even before the program’s entrance into the local context. The first issue is coherent with international literature [38], according to which management decisions reign supreme in reducing barriers and promoting strategic facilitation, even more so in the case of SFP 10–14, where, in Brazil, the program is implemented via public policy. Management barriers such as late payments and bureaucratic impositions in letting professionals participate in the program training, were reflected in the engagement of group facilitators and, consequently, in the delivery of SFP 10–14. As for the second item, the lack of intersectoral coordination in the initial stages of the program impeded distributing tasks among group facilitator from different services, increasing workload of some group facilitators. Besides, the lack of intersectoral coordination also hampered family recruitment since network professionals, who serve different audiences, could have recruited families better fitted for SFP 10–14 profile.

Intersectorality also played a prominent role as a barrier, though less so than the its facilitative role. Interviewees discourse evinced difficulties with operationalizing intersectoral actions even before the arrival of SFP 10–14, which suggest the need to improve coordination between assistance, health care, and education before the municipality adopts the program. When appropriate, intersectorality reduced common barriers to program planning, allowing for better distribution of tasks and, consequently, a reduced overload for the professionals.

Likewise, the working conditions of the professionals, particularly in team training and SFP 10–14 delivery stages, was highlighted as a barrier. The workload in their existing activities (outside of SFP 10–14) left little time for planning. Similarly, Patterson [39] pointed out that the overload and long working hours increased the risk of reduced work performance errors related to fatigue. Another study that analyzed implementation barriers and facilitators for a community-based program identified professional implementors as having a greater sense of belonging and engagement with a program when they didn’t have to divide their time between its implementation and other activities [40].

Practical implications can be extracted from the present study. First, to improve the implementation quality of SFP 10–14 in Brazil, public administration would need to be committed to the costs demanded by the programming planning and execution, including those related to the group facilitators’ working conditions, which was considered one of the main challenges to engagement and to logistical organization in the program’s execution. Second, improvements in the intersectoral coordination are needed in order to reduce obstacles to program planning, enhance task distribution, and lower the professionals’ workload. Third, implementation strategies could be strengthened by teaching skills for managing groups, the intervention’s change mechanisms, and strategies for reaching the target audience to implementation agents. Finally, monitoring and supervision should be maintained, as the reports indicate that doing so reduced errors arising from the team training stage and impacted group facilitator self-efficacy and delivery quality of the program [41].

The present study, to our knowledge, pioneered the systematic examination contextual elements influential to the implementation of SFP 10–14 as a public policy tool. It was innovatively based on the CICI model, that has the advantage of providing a detailed and broad description of context phenomenon and its relationship with implementation of complex interventions. This model has shown to be compatible with the research question of this study, by allowing the understanding of how much the context has an influence on the successful outcomes of a policy or program, such as the SFP 10–14. Furthermore, it enabled an objective categorization of the observed events. In addition to this, the study’s results expand the findings Latin American studies already available, where there is a paucity of research on the intervention’s implementation and effectiveness [20]. According to the present study’s findings, potentially risky SFP 10–14 implementation pathways would be characterized by an unsupportive political context, unengaged public administration agents, implementation agents and services with a lack of organizational capacity to carry out the implementation, and implementation strategies with deficient investment in intersectoral coordination and group facilitators’ training. Instead, potentially safer implementation paths would be characterized by offers of political support, a prevention culture present in the political agenda, engaged administration agents, well-trained implementation agents with good working conditions, and an integrated intersectoral network with embedded professionals.

Limitations can be seen in this study. It is assumed that the results found would be on firmer ground if triangulated from the perspective of the group facilitators, professionals who work with SFP 10–14 more actively starting from the team training stage. Furthermore, this research did not use methods of data collection other than interviews and focus groups. Therefore, the typical social desirability of verbal measures constitutes an important bias that should be considered when interpreting these findings. Finally, the data was collected at a specific point in time, in a cross-sectional analysis, which may mask historical subtleties that could be revealed in a longitudinal or ethnographic design with longer and deeper field immersion.

## 5. Conclusions

This study’s findings suggest the SFP 10–14 implementation was perceived as onerous and complex, with barriers being reported with slightly higher frequency than facilitators, and whose viability requires a favorable political context, engaged implementation agents, and carefully-designed implementation strategies. Investment in the various preimplementation and implementation phases should be made in management, key actor awareness, and implementation strategy quality. The results highlight the importance of examining organizational and community readiness to verify the conditions necessary for SFP 10–14 preimplementation and implementation in the public sector, or risk it being impractical [42].

Future studies could analyze to what degree favorable or unfavorable SFP 10–14 implementation contexts impacted its effectiveness and sustainability in states where it was implemented as an integral part of the National Drug Policy. Therefore, study designs capable of covering the interrelationships between the result patterns, mechanisms, and contexts would represent an important contribution to the area by uncovering the circumstances that explain the effectiveness of SFP 10–14 or its absence [43]. Similarly, analyses of deimplementation, on the one hand, or sustainability, on the other, associated with the context and causal mechanisms are suggested. These findings could be turned into inputs for training implementation agents as well as have broad applicability in studies of local preventive technologies and the translation of the interventions into public policies, especially in resource-scarce contexts.

## Figures and Tables

**Table 1:** Expected outcomes for each stage of the SFP 10–14 action theory in Brazil.

Action Theory Stages	Expected Outcomes
Negotiation for local adoption of SFP 10–14	Negotiation between federal and municipal spheres for the coming of SFP 10–14 to the states. A technical-operational agreement seals an initial implementation. The recommendation of a local coordinator and intersectoral adoption is expected, with consensus of tasks from each sector involved.
Local coordination	Hiring and instruction of federal trainers. Federal trainer integration with local team. Elaboration of a training schedule to program articulator and multiplier to operationalize the implementation.
Services mobilization	Adoption of SFP 10–14 by professionals and services that will effectively deliver the program and strengthen the support the SFP 10–14 implementation. Building of organizational and community support by the municipal articulator.
Team training	Implementation agents trained in the paradigm governing the National Drug Policy and the SFP 10–14 objectives, bases, and methodology.
Family recruitment	Advertisement to, invitation of, and selection of SFP 10–14 participant families. It includes strategies to gather groups of seven to ten families to start the program as well as their initial engagement.
Planning of implementation logistics	Planning and organization of infrastructure, materials, and procedures required for implementation.
SFP 10–14 delivery	Implementation of the seven meetings with parents/legal guardians, youths, and families. Weekly session planning and monitoring in order to correct implementation problems.

**Table 2:** Definitions of inductive analysis categories.

Categories	Definitions
Infrastructure	Material and resources needed for implementation.
Prevention culture	Identification by public agency administrators, the professionals and institutions that shares values, objectives, and methodologies of SFP 10–14, which may be the result of experience with previous iterations or another experience in prevention.
Financial resources	The capital needed to implement SFP 10–14.
Municipal management	Management actions related to the management of people in their activities, financial resources, and the intersectoral policy.
Group facilitators’ working conditions	Aspects of workload, time availability, number of people available to perform required activities, work accumulation, sensation of job instability, and employment precarity.
Institutional disruption	Refers to the period of political instability in 2015 that culminated in 2016 with the impeachment of President Dilma Rousseff and her removal from the Presidency of the Republic.
Intersectorality	Coordination via agreement among various social public policies aiming to implement the program.
Corruption	Diversion of funds.
Elections	Political agreements and campaign support during the election period, August through October, every even numbered year in the municipalities.
Awareness of hierarchical flow	Understanding that awareness occurs according to hierarchical flows of power, in which a higher-level manager is responsible for raising awareness of lower-level managers.
Federal supervision	Decisions concerning SFP 10–14 implementation logistics in the municipality together with federal and municipal service leaders, including the provision of implementation kits and the hiring of federal trainers.
Aggregate value of SFP 10–14	Benefits gained from experience in program implementation.
Local peculiarities	Strikes and holidays occurring during the implementation.
Selection of public service	Strategies used by federal trainers in selecting where to implement SFP 10–14.
Selection of families	The criteria established for selecting families to participate in the program.
Group facilitator training methodology	Strategies and methodologies used to train program facilitators.
Approach for inviting families	Strategies used to disseminate the program and information offered to families invited to participate in SFP 10–14.
Family maintenance strategies	Strategies used in the awareness-raising and recruitment meetings and weekly sessions to boost family adherence.
Federal support and monitoring in the implementation	Operational support by federal trainers for group facilitators during the SFP implementation
Planning	Holding weekly planning meetings during the SFP 10–14 implementation, where activities to be carried out with families are discussed, materials for implementation organized, and the best session management strategies verified.
Compatibility of meeting and family schedules	Reconciliation of session times between professionals and families according to availability of both groups.
Perception of effects on families	The perception of positive effects on the families as an essential element for motivation of the program facilitator.
Prior relationship between service and community	The closeness of the relationship between the service implementing the program and the individuals from the community who constitute the SFP 10–14 target audience.
Awareness-raising of public agency administrator	Information provided by federal trainers to public agency administrators about the program and strategies used by trainers to mobilize them in favor of implementing SFP 10–14.
Awareness-raising of group facilitators	Information provided by federal trainers, public agency administrators, or program multipliers to group facilitators aiming to mobilize them in favor of implementing SFP 10–14.
Team training awareness-raising	The understanding that professionals need to receive information about the real complexity of implementing SFP 10–14 before going through the facilitator team training.
Adherence of professionals	Engagement of public agency administrators, as well as health care, education, and social service professionals in the SFP 10–14 implementation and the quality of their participation in program delivery.
Adherence of institutions	Engagement by institutions as a whole, not the individuals comprising them, whether departments of education or health care, or other government agencies.
Professionals’ skills	Professionals with attitudes and actions that exceed expectations regarding the exercise of their function and that favor the implementation.
Relationships between professionals	Relationship and communication quality between professionals involved in the SFP 10–14 implementation process.

**Table 3:** Definitions of the CICI model categories (Pfadenhauer et al., 2017).

Elements	Definitions
Geographic context	The physical and resource aspects of the environment, both natural and human-transformed (e.g., infrastructure), which are available in a given space.
Epidemiological context	The disease network as well as the determinants of the population’s needs, including demography.
Sociocultural context	Comprises explicit and implicit behavior patterns, including their incorporation in the form of symbols. The essential culture core consists of ideas and values historically derived and selected that are shared by the members of a group. It refers not only to the conditions under which people are born, grow, live, and work or their ages, but also the social roles a human being assumes as a member of a family, of a community, or as a citizen, as well as the relationships inherent in these roles. Constructs such as knowledge, beliefs, concepts, customs, institutions and any other capacities and habits acquired by a group are included in this context.
Socioeconomic context	The social and economic resources of a community and its access by the population.
Ethical context	Moral reflections that encompass the norms, rules, patterns of conduct, and principles that guide the decision-making and behavior of individuals and institutions. The ethical, sociocultural, and legal aspects are strongly interrelated.
Legal context	The rules and regulations established to protect the rights of the population and the interests of society. Formally, these should be approved by a competent legislative body, such as a congress. The legal norms may be applied by order and coercion, which distinguishes them from ethical and social ones.
Political context	The distribution of power, resources, and interests among the population as a whole, as well as among organizations according to formal rules or information from the interactions between them. Also, the accessibility to the location offering the service, such as service delivery, leadership and governance, health information, human resources, and financing. Strongly related to the socioeconomic and sociocultural elements of the population in question.
Implementation theory	The implementation of a program theory seeks to explain the causal mechanisms of the implementation; it is, thus, analogous to a program theory, which explains the causal mechanisms that connect an intervention to its results. An implementation theory formalizes how a change needs to be executed so that the implementation effort is successful. Additionally, it serves as the basis for the implementation process and strategies.
Implementation process	The whole social process by which the implementation is operationalized. It relies on the tactics and methods used by change-promoting leaders. It is a multistage process that does not necessarily take place linearly. At some points, it is expected that the implementation agents will capture corrections, refinements, or expansions. The steps range from the evaluation of needs and community resources to agent awareness, program adoption, preparation, and changes necessary for implementation. The team should be well-informed about the program and information should be disseminated. This follows a pilot or initial implementation in which the intervention may be readjusted to accommodate the community’s daily routine, thus making it more sustainable.
Implementation strategy	The set of activities chosen and customized to fit the program theory in its delivery context. These strategies may change over time and they ensure the adoption of the program and future sustainability. This takes place prior to the actual implementation and refers to planning strategies and awareness of the implementation process.
Implementation agents	These are individuals who possess a combination of attributes, knowledge, skills, beliefs, positions, and attitudes that exercise significant influence on the implementation. Implementation agents could be either local public agency administrators or frontline professionals aware of and skilled in the implementation. The agents may also be recipients of the implementation or institutions when these entities play a primary role in the implementation.

**Table 4:** Frequency of topics identified as facilitators of the SFP 10–14 implementation process by the action theory stage in Brazil.

	SFP 10–14 Action Theory Stages						
Categories	Negotiation for Local Adoption of SFP 10–14	Local Coordination	Services Mobilization	Team Training	Family Recruitment	Planning of Implementation Logistics	SFP 10–14 Delivery
Approach for inviting families					14		
Adherence of professionals	4		4	2		3	6
Adherence of Institution	1		1				1
Compatibility of meeting and family schedules							1
Group facilitator working conditions	1			1			1
Prevention culture	9	1		1			1
Selection of public service		5			1		2
Family maintenance strategies					7		3
Awareness of hierarchical flow	2		5				
Municipal management	2	2	4			2	4
Professionals’ skills				2	2		
Infrastructure			2	1		2	2
Intersectorality	7		3	1	4	7	1
Group facilitator training methodology				11			
Perception of effects on families							8
Planning						8	3
Financial resources							1
Relationships between professionals						1	4
Prior relationship between the service and the community					6		2
Public agency administrator awareness	8	1	6				1
Group facilitator awareness		1	4		4		
Team training awareness-raising	1		5				
Federal supervision	1						3
Federal support and monitoring in implementing the SFP 10–14			2				4
SFP 10–14 aggregate value				1			
Total	**36**	**10**	**39**	**20**	**38**	**23**	**48**

**Table 5:** Frequency of topics pointed out as barriers to the SFP 10–14 implementation process by the stage of the action theory in Brazil.

	SFP 10–14 Action Theory Stages						
Categories	Negotiation for Local Adoption of SFP 10–14	Local Coordination	Services Mobilization	Team Training	Family Recruitment	Planning Implementation Logistics	SFP 10–14 Delivery
Adherence of professionals	2		5	3			3
Adherence of Institutions	1		5				1
Compatibility of meeting and family schedules					2	5	
Group facilitator working conditions	1	1	12	4			13
Corruption						1	
Prevention culture	1	2	2	1	1	1	3
Elections	1					1	
Selection of families					4		6
Selection of public service		1					1
Awareness of hierarchical flow	1						
Municipal management	5	3	11			3	10
Infrastructure		1				15	6
Intersectorality	3		3		1		3
Group facilitator training methodology				10			7
Location peculiarities		1	3	1			3
Planning						2	4
Financial resources	1			2		5	5
Relationships between professionals	2		2		1	1	2
Institutional rupture	1		2	1			
Public agency administrator awareness			3				
Group facilitator awareness	1	1		2	4		1
Team training awareness-raising		1	7				
Federal supervision		2					
Federal support and monitoring in implementing the SFP 10–14							1
SFP 10–14 aggregate value			1				
Total	**25**	**13**	**56**	**24**	**13**	**34**	**69**

**Table 6:** Combination of inductive and deductive categories.

Elements	Categories
Geographic context	Infrastructure
Epidemiological context	N/A
Sociocultural context	Prevention culture
Socioeconomic context	Financial resources
Ethical context	N/A
Legal context	N/A
Political context	Municipal management Group facilitators’ working conditions Institutional disruption Intersectorality Corruption Elections Awareness of hierarchical flow Prior relationship between service and community Federal supervision
Implementation theory	N/A
Implementation process	Aggregate value of SFP 10–14 Local peculiarities
Implementation strategy	Group facilitator training methodology Approach for inviting families Family maintenance strategies Federal support and monitoring in the implementation Planning Selection of public service Selection of families Perception of effects on families Compatibility of meeting and family schedules
Implementation agents	Awareness-raising of public agency administrator Awareness-raising of group facilitators Professionals’ skills Adherence of professionals Adherence of institutions Team training awareness-raising Relationships between professionals
